# The role of state breastfeeding laws and programs on exclusive breastfeeding practice among mothers in the special supplemental nutrition program for Women, Infants, and Children (WIC)

**DOI:** 10.1186/s13006-022-00490-9

**Published:** 2022-06-25

**Authors:** Paschal A. Apanga, Elizabeth J. Christiansen, Ann M. Weber, Lyndsey A. Darrow, Mark S. Riddle, Wei-Chen Tung, Yan Liu, Taya Kohnen, Joshua V. Garn

**Affiliations:** 1 School of Public Health, University of Nevada, Reno, 1664 N Virginia St, SMS suite 102, NV 89557 Reno, USA; 2 School of Medicine, University of Nevada, Reno, 1664 N Virginia St, NV 89557 Reno, USA; 3 The Valley Foundation of School of Nursing, San Jose State University, 1 Washington Sq, San Jose, CA 95192 USA

**Keywords:** Breastfeeding, Exclusive breastfeeding, Laws, Women, Infants, Children, Effect modification

## Abstract

**Background:**

It is unclear if state laws supporting breastfeeding are associated with exclusive breastfeeding (EBF) practice among low-income mothers participating in the Special Supplemental Nutrition Program for Women, Infants, and Children (WIC). The main objectives of our study were to assess the relationship between such laws and EBF among WIC-participating mothers and to assess whether this association varied by employment status. We also assessed how mother’s exposure to WIC breastfeeding consultation was associated with EBF.

**Methods:**

A cross-sectional study was conducted across seven WIC program locations (i.e., Georgia, Massachusetts, Nevada, Pennsylvania, Wisconsin, Wyoming, Chickasaw Nation) between July–August 2020. Data were collected using convenient sampling from each program location and surveys were administered electronically or on paper to WIC-participating mothers. We restricted our analysis to data from 1161 WIC-participating mothers with infants aged zero to five months old. Multivariable mixed models were used to estimate the relationship between our exposures of interest (i.e., number of laws supporting breastfeeding, employment-related breastfeeding laws, WIC breastfeeding consultation) and EBF, while controlling for potential confounders and accounting for clustering by program location. Effect modification by employment status was assessed on the additive and multiplicative scales.

**Results:**

Among WIC-participating mothers living in program locations with no employment-related breastfeeding laws, EBF was 26% less prevalent for employed mothers compared to unemployed mothers (adjusted prevalence ratios [aPR]: 0.74, 95% CI: 0.67,0.83). Among all mothers, a one-unit increase in laws supporting breastfeeding was not associated with EBF (aPR: 0.88, 95% CI: 0.71,1.10). However, among employed mothers, living in areas with more employment-related laws was associated with a higher prevalence of EBF (aPR: 1.43, 95% CI: 0.83, 2.44). Infants whose mothers received a WIC breastfeeding consultation had 33% higher prevalence of being exclusively breastfed compared to infants whose mothers did not receive a WIC breastfeeding consultation (aPR: 1.33, 95% CI: 1.05,1.70).

**Conclusions:**

Infants whose WIC-participating mothers were employed, were less likely to be exclusively breastfed, but our effect modification analyses showed that laws supporting breastfeeding at the workplace may promote EBF among employed women. EBF was more prevalent among mothers who received a WIC breastfeeding consultation compared to those who did not receive such a consultation.

**Supplementary Information:**

The online version contains supplementary material available at 10.1186/s13006-022-00490-9.

## Background

Breastfeeding is an important practice that provides nutrients for the optimal growth and development of the newborn [1]. Breastfeeding protects infants from adverse effects of poor nutrition to enable them to obtain their full growth potentials [2]. The American Academy of Pediatrics affirms the importance of breastfeeding, and recommends six months of exclusive breastfeeding (EBF), followed by continued breastfeeding as complementary foods are introduced, with continuation of breastfeeding for one year or longer as desired by mother and infant [3].

Though breastfeeding initiation and duration has improved among mothers in the US over time, 60% of women do not breastfeed for as long as they intend to [4]. While 83.9% of infants in the United States (US) have ever been breastfed, only 25.8% of infants born in 2018 were exclusively breastfed for 6 months, and 35.0% of infants were breastfeed for 1 year [5]. The low breastfeeding prevalence has been attributed to a number of physical/medical and social barriers. Physical/medical barriers include inadequate milk supply, breast pain and lactation problems, Cesarean sections [6], and that breastfeeding is time intensive, which may make it difficult for mothers to practice EBF in the workplace [7]. Social barriers that prevent mothers from meeting their breastfeeding goals include lack of support from employers and child care facilities, poor family and social support, lack of knowledge about breastfeeding, feeling embarrassed to breastfeed in public places, and lack of peer support during breastfeeding [6]. Overcoming barriers to breastfeeding in the US is essential, particularly in meeting the Healthy People 2030 goal of increasing the proportion of infants who are exclusively breastfed through age 6 months to 42.4% [8].

A number of US laws have been enacted at the state or federal levels supporting breastfeeding and potentially addressing some of the aforementioned barriers. The enactment of the federal law on Patient Protection and Affordable Care Act in 2010 requires employers to provide “reasonable break time” and space to support breastfeeding mothers [9]. However, this law covers only employees who work for hourly wages, allows mothers only to express breast milk, and exempts small businesses [9, 10]. Some working mothers have sought legal protection using federal laws in order to breastfeed in the workplace, but have had little success in securing the ability to breastfeed [9]. Most laws supporting breastfeeding in the US are enacted at the state level [9, 11]. State laws encourage breastfeeding in the workplace, and prohibit employers from discriminating against breastfeeding employees. These laws also exempt mothers who breastfeed from public indecency laws and from jury duty, and protect them to breastfeed in any public or private space [11]. Hawkins et al. observed that state laws supporting breastfeeding appear to increase breastfeeding rates, while Kogan and his colleagues found that children in states without breastfeeding laws were associated with higher odds of not being breastfeed [10, 12]. State laws supporting breastfeeding, which include having break time and having a private space at the workplace to breastfeed, have been associated with mothers being more likely to breastfeed for six months [13]. However, the association between laws supporting breastfeeding and EBF has not been well characterized among low-income women living in the USA and participating in the Special Supplemental Nutrition Program for Women, Infants, and Children (WIC) program.

WIC is a nutrition assistance program that safeguards the health of low-income pregnant and post-partum women, breastfeeding women, infants, and children younger than five years by providing nutritious foods to supplement diets, information on healthy eating, and referrals to seek health care [14]. The program started as a pilot program enacted by the Child Nutrition Act of 1966 and now serves nearly one half of all infants born in the US [14]. One of the key priorities of the program is to support breastfeeding among participating mothers and infants in accordance with the recommendation of the American Academy of Pediatrics so that mothers and children can benefit from the short- and long-term health benefits of breastfeeding [3, 14]. While some studies observed lower prevalence of breastfeeding practices among WIC mothers compared to eligible non-WIC mothers [15, 16], others have found a positive relationship between WIC mothers and breastfeeding rates [17, 18]. We use the term “WIC mothers” to refer to women who participate in WIC, for example, by receiving nutritious foods, referrals and counseling.

Our goal in this study was to estimate the strength of association between WIC program locations’ (i.e., Georgia, Massachusetts, Nevada, Pennsylvania, Wisconsin, Wyoming, Chickasaw Nation) breastfeeding laws and WIC mothers EBF prevalence, and to assess whether the relationship with employment-related laws was modified by employment status. We hypothesized that WIC mothers living in program locations that have laws supporting breastfeeding are more likely to report EBF compared to WIC mothers in locations with fewer breastfeeding laws. We also assessed how mothers’ exposure to WIC breastfeeding consultation is associated with EBF, and assessed whether this relationship is modified by employment status. We hypothesized that mothers who received a WIC breastfeeding consultation (i.e., receiving breastfeeding information from a WIC staff member, lactation consultant or peer counselor) are more likely to practice EBF compared to WIC mothers who did not receive this information, and that this relationship will be modified by employment status.

## Methods

### Study design, data collection and study population

This was a cross-sectional study conducted in seven WIC program locations in six states (i.e., Georgia, Massachusetts, Nevada, Pennsylvania, Wisconsin, Wyoming) and in one Indian Tribal Organization (ITO; Chickasaw Nation, located in Oklahoma) between July 2020 and August 2020. In the overarching study, data were collected among WIC mothers of young children and expectant mothers in their third trimester aged 18 years old or above as part of a baseline survey of a one-year breastfeeding campaign in the six states and the ITO [19]. The campaign builds on the proven strengths of a social marketing approach for breastfeeding promotion among WIC mothers. The campaign interventions include social marketing activities, the buddy program (a program that matches breastfeeding WIC mothers or expectant mothers whose babies were born or will be born around the same date to share experiences, encourage each other to breastfeed, and celebrate their children’s milestones) [20], and WIC clinic staff education. However, in this study, we restricted our study population to WIC mothers with infants aged 5 months old or less, as this is the age group of infants who are recommended to be exclusively breastfed according to the American Academy of Pediatrics [3].

#### Sampling strategy

Participants for the study were recruited using a convenient sampling approach. The WIC staff in the six states and the ITO administered the surveys either electronically through an online survey application or on paper to WIC-participating mothers. Responses to both online and paper surveys were de-identified. WIC staff members in each program location kept the list of WIC participants numbers onsite and assigned a project ID to participants for use on the surveys. While responses to the online survey application went directly into a survey application, responses to the paper surveys were entered into the survey application by WIC staff. Data were collected on self-reported intention to breastfeed (but reported after birth), breastfeeding-related practices, obstetric characteristics, and socio-demographics. Data also were collected on breastfeeding services and resources available through the WIC program.

### Primary outcome

The primary outcome of interest is EBF, which was defined as the mother feeding her infant only breastmilk up through 5 months of age.

### Predictors

The predictors of interest in our study include laws supporting breastfeeding and WIC breastfeeding consultation. Employment status was assessed as an effect modifier.

Laws related to the promotion of breastfeeding in each state or ITO were assessed by extracting data from the US National Conference of State Legislatures (NCSL) on breastfeeding laws, which provides a summary of breastfeeding laws from 50 states, the District of Columbia, Puerto Rico and the Virgin Islands, as of September 2020 [21]. Program coordinators confirmed that laws in Chickasaw Nation were similar to those in Oklahoma, the state that surrounds Chickasaw Nation on three sides. We categorized laws supporting breastfeeding into five themes: (1) employers are encouraged or required to provide break time and private space for breastfeeding employees; (2) employers are prohibited from discriminating against breastfeeding employees; (3) breastfeeding is permitted in any public or private location; (4) breastfeeding is exempt from public indecency laws; and (5) breastfeeding women are exempt from jury duty (Additional file 1) [11]. The number of laws supporting breastfeeding in each state or ITO were summed to create a continuous score (i.e., a score of 1 for each theme and the total score range from 1 to 5). Breastfeeding laws in themes (1) and (2) were grouped together as employment-related laws. A binary indicator was created for whether the states or ITO had any employment-related breastfeeding laws or not. WIC breastfeeding consultation was defined as mothers who self-reported that they received breastfeeding information from a WIC program staff member, lactation consultant or peer counselor (yes, no). Employment status was also self-reported, and we dichotomized employment into any employment versus no employment. Mothers were considered employed if they reported being employed full-time or part-time.

### Covariates

There were both individual- and program-level covariates. Individual-level factors include mother’s age (years), education (< high school graduate, ≥ high school graduate), marital status (married/living with partner, single), has an adult support for baby at home (yes, no), current smoker (yes, no), and infant age (< 8 weeks, 8–16 weeks, > 16 weeks). Other individual-level factors include the mother’s previous history of breastfeeding (yes, no), parity (primiparity, multiparity), infant was delivered by cesarean section (yes, no), and infant was born premature (yes, no). Program-level factors include whether the mother received any breastfeeding promotional messages (yes, no), and whether she received breastfeeding information from any breastfeeding support group (yes, no).

### Data analysis

Descriptive statistics of the study population were stratified by program locations. The overall prevalence of categorical variables, and the means and standard deviations of continuous variables are presented separately for each of the program locations.

We used four separate multivariable mixed models to conduct our analyses. Each of these models used modified Poisson regression with a robust variance estimator to estimate the prevalence ratio, and accounted for clustering by program location using a random intercept.

The first model assessed the relationship between the number of laws supporting breastfeeding in each program location and EBF, while adjusting for potential confounders, which include WIC breastfeeding consultation, individual- and program-level factors. Biologically plausible potential confounders were specified a priori and were controlled for in the model (Additional file 2).

The second model was used to estimate the relationship between program location’s employment-related breastfeeding laws and EBF practice. We introduced an interaction term (program location’s employment-related breastfeeding laws*employment status) in the model and assessed effect modification by employment status on the additive and multiplicative scales, while adjusting for the number of laws supporting breastfeeding, WIC breastfeeding consultation, individual-and program-level factors.

The third model assessed the association between WIC breastfeeding consultation and EBF practice, while adjusting for the number of laws supporting breastfeeding, employment status and individual- and program-level factors. The fourth model was similar to the third but included an interaction term between WIC breastfeeding consultation and employment status (WIC breastfeeding consultation*employment status). Effect modification was assessed on the additive and multiplicative scale, while adjusting for the same variables as in model 3.

Effect modification on the additive scale was assessed using the relative excess risk due to interaction (RERI). The additive scale can reliably identify the correct group of individuals to intervene in order to achieve a significant public health impact [22, 23]. Therefore, it is the most appropriate public health measure for assessing effect modification on the additive scale. RERI is an extra risk due to interaction, which is estimated as the difference between the (expected) effect based on the summation of the separate effects of the two predictors under study and the (observed) effect in the joint exposure category [24]. When RERI is equal to zero, it means no interaction, while a RERI greater than zero means positive interaction, and a RERI less than zero means negative interaction [25]. The RERI and corresponding 95% confidence intervals were estimated using publicly available SAS code [26]. On the multiplicative scale, an estimate of one means no effect modification, and an estimate of less than one signifies negative effect modification and an estimate greater than one signifies positive effect modification [27]. Our effect modification results were presented in a tabular format recommended by Knol and VanderWeele [27].

While we did not assess the role of each of the specific breastfeeding laws on EBF, we did conduct sensitivity analyses to compare the prevalence of EBF among mothers in program locations with laws encouraging or requiring employers to provide break time and private space for breastfeeding employees to mothers in program locations without such laws. We also compared the prevalence of EBF among mothers in program locations with laws that prohibit employers from discriminating against breastfeeding employees to mothers in program locations that do not prohibit such discrimination. We conducted sensitivity analyses to see whether the relationship between the number of laws supporting breastfeeding and EBF remained the same across different infant age (0–1, 2–3 & 4–5 months) groups. We also conducted sensitivity analysis to assess the relationship between a 10-unit increase in number of law-years in each program location and EBF. Number of law-years was defined as the number of total years of laws enacted in each state (Additional file 1). Data analyses were conducted using SAS version 9.4 (SAS Institute, Cary, NC).

## Results

Our study population consisted of 1161 mothers with infants 5 months of age or less (Table 1). The mean age of mothers and infants across seven program locations ranged from 19–27 years and 3–13 weeks, respectively (Table 1). The majority of mothers had received a WIC breastfeeding consultation across all program locations. Similarly, most mothers had at least a high school level of education across all program locations. Caucasian/White mothers were majority in three of the program locations (Chickasaw Nation, Pennsylvania, Wyoming), while Hispanic mothers were more prevalent in two program locations (Massachusetts, Nevada). Black/African mothers were majority in Georgia and Wisconsin. The prevalence of EBF ranged from 20% in Massachusetts to 69% in Wyoming. Mothers who reported any current breastfeeding ranged from 37% in Pennsylvania to 88% in Wyoming (Table 1). There was also little missing information in our data (Table 1).

**Table 1:** Characteristics of mothers participating in WIC in seven program locations (*n* = 1161)

**Characteristics**	CN*	GA*	MA*	NV*	PA*	WI*	WY*
Total sampled	103	88	89	524	256	12	89
**Socio-demographic factors**							
Mother's age (years), mean (*SD)	25 (5)	26 (25)	23 (28)	27 (14)	27 (10)	19 (37)	27 (5)
Infant’s age (weeks), mean (SD)	13 (8)	12 (9)	3 (2)	11 (8)	7 (4)	8 (11)	11 (8)
Infant’s age (%)							
< 8 weeks	34 (33)	33 (37)	85 (96)	216 (41)	130 (51)	8 (67)	38 (43)
8–16 weeks	26 (25)	19 (22)	4 (4)	133 (25)	123 (48)	1 (8)	23 (26)
> 16 weeks	43 (42)	36 (41)	0	175 (33)	3 (1)	3 (25)	28 (32)
Education (%)							
< High school graduate	16 (16)	7 (8)	21 (26)	99 (20)	49 (20)	2 (17)	15 (17)
≥ High school graduate	83 (84)	78 (92)	59 (74)	409 (80)	196 (80)	10 (83)	74 (83)
missing	4	3	9	16	11		
Marital status (%)							
Married/living with partner	67 (68)	42 (49)	43 (54)	317 (62)	128 (53)	9 (75)	63 (71)
Single	32 (32)	43 (51)	37 (46)	191 (38)	115 (47)	3 (25)	26 (29)
missing	4	3	9	16	13		
Employment status (%)							
Unemployed	62 (63)	54 (64)	57 (71)	380 (75)	154 (63)	6 (50)	60 (67)
Employed	37 (37)	30 (36)	23 (29)	128 (25)	89 (37)	6 (50)	29 (33)
missing	4	4	9	16	13		
Race/Ethnicity (%)							
Caucasian/White	36 (36)	3 (4)	21 (27)	102 (21)	198 (86)	2 (18)	66 (76)
Asian/Pacific Islander	0	6 (8)	2 (3)	35 (7)	1 (0)	2 (18)	0
Black/African American	2 (2)	54 (69)	7 (9)	50 (10)	15 (7)	4 (36)	0
American Indian/Alaska Native	28 (28)	0	0	6 (1)	0	0	2 (2)
Hispanic/Latina	6 (6)	11 (14)	39 (49)	249 (51)	4 (2)	2 (18)	14 (16)
Multiracial	27 (27)	4 (5)	5 (6)	46 (9)	11 (5)	1 (9)	4 (5)
Other	0	0	5 (6)	2 (1)	2 (1)	0	1 (1)
missing	4	10	10	34	25	1	2
Has an adult support for baby (%)	80 (81)	54 (64)	52 (64)	357 (70)	168 (69)	9 (75)	67 (75)
missing	4	3	8	16	13		
Current smoker (%)	11 (11)	5 (6)	7 (7)	14 (3)	44 (18)	1 (8)	4 (5)
missing	4	3	8	16	11		
**Obstetric factors**							
Parity (%)							
multiparity	57 (55)	57 (65)	57 (64)	353 (67)	165 (64)	12 (100)	56 (63)
primiparity	46 (45)	31 (35)	32 (36)	171 (33)	91 (36)	0	33 (37)
Cesarean section (%)	34 (33)	32 (36)	29 (35)	145 (28)	82 (32)	6 (50)	21 (24)
missing			5	3			
Prematurity (%)	12 (12)	15 (17)	18 (21)	43 (8)	28 (11)	3 (25)	8 (9)
missing			5	3			
**Breastfeeding-related factors**							
Exclusive breastfeeding (%)	32 (31)	27 (31)	17 (20)	202 (39)	57 (22)	5 (42)	61 (69)
missing			2	3			
Currently breastfeeding (%)	51 (50)	73 (83)	56 (63)	429 (82)	95 (37)	8 (67)	78 (88)
Ever breastfeed (%)	88 (85)	86 (98)	71 (80)	510 (98)	173 (68)	9 (75)	87 (98)
missing				1			
Received breastfeeding promotion (%)	91 (88)	71 (81)	62 (70)	418 (80)	202 (79)	9 (75)	68 (76)
History of breastfeeding (%)	44 (43)	46 (52)	40 (46)	304 (58)	106 (41)	10 (83)	51 (57)
missing			1				
Received information from any breastfeeding support group (%)	23 (23)	29 (33)	12 (14)	102 (20)	42 (17)	4 (33)	17 (19)
missing			4	3	4		1
WIC breastfeeding consultation (%)	97 (95)	66 (75)	68 (80)	469 (90)	208 (83)	10 (83)	76 (86)
missing	1		4	3	4		1

*CN Chickasaw Nation, *GA Georgia, *MA Massachusetts, *NV Nevada, *PA Pennsylvania, *WI Wisconsin, *WY Wyoming, *SD Standard deviation

Among all mothers, there was no relationship between a one-unit increase in laws supporting breastfeeding and EBF (adjusted prevalence ratio [aPR]: 0.88, 95% CI: 0.71,1.10; Table 2). A number of individual-and program-level variables were associated with EBF among all mothers (Table 2). We found that Hispanic/Latina women had 35% lower prevalence of EBF compared to Caucasian/White women (aPR: 0.65, 95% CI: 0.46,0.92). Mothers who smoke had 38% lower prevalence of EBF compared to their peers that were not smoking (aPR: 0.62, 95% CI: 0.44,0.87). EBF was also more prevalent among primiparous women compared to multiparous women (aPR: 2.12, 95% CI: 1.43,3.12). In addition, EBF was 20% less prevalent among mothers that had a Cesarean section compared to mothers that had a vaginal delivery (aPR: 0.80, 95% CI: 0.74,0.87). Infants whose mothers had a previous history of breastfeeding and received breastfeeding information from any support group were 2.71 and 1.30 times more likely to be exclusively breastfed, respectively, compared to infants whose mothers had no prior history of breastfeeding and did not receive breastfeeding information from any support group (Table 2).

**Table 2:** The association between number of breastfeeding laws and EBF practice (Model I^a^)

Variable	^b^Adjusted PR (95% CI)
**Main exposure of interest**	
Number of breastfeeding laws	0.88 (0.71,1.10)
**Potential confounders**	
No	Reference
WIC breastfeeding consultation	1.33 (1.05,1.68)
No	Reference
Has an adult support for baby at home	1.03 (0.90,1.19)
No	Reference
Current smoker	0.62 (0.44,0.87)
Parity	
multiparity	Reference
primiparity	2.12 (1.43,3.12)
Vaginal delivery	Reference
Cesarean section	0.80 (0.74,0.87)
Term baby	Reference
Premature baby	0.82 (0.62,1.10)
No	Reference
Received breastfeeding promotional messages	1.04 (0.79,1.36)
No	Reference
Previous history of breastfeeding	2.71 (1.89,3.88)
No	Reference
Received breastfeeding information from a breastfeeding support group	1.30 (1.11,1.52)
Mother's age (years)	1.00 (0.99,1.01)
Infant’s age	
< 8 weeks	Reference
8–16 weeks	1.11 (0.96,1.29)
> 16 weeks	1.07 (0.92,1.25)
Education	
< High school graduate	Reference
≥ High school graduate	1.01 (0.72,1.41)
Marital status	
Married/living with partner	Reference
Single	0.94 (0.83,1.06)
Race/Ethnicity	
Caucasian/White	Reference
Asian/Pacific Islander	0.67 (0.36,1.22)
Black/African American	0.81 (0.58,1.13)
American Indian/Alaska Native	1.05 (0.78,1.40)
Hispanic/Latina	0.65 (0.46,0.92)
Multiracial	0.81 (0.52,1.26)
Other	2.00 (0.86,4.68)

^a^Model I adjusted for all the variables shown in the table and also adjusted for clustering

^b^Adjusted PR is adjusted prevalence ratio

Our analysis on effect modification by employment status on the relationship between employment-related breastfeeding laws and EBF, found the estimate was in the direction that supports a higher EBF prevalence among employed mothers, although the confidence intervals included the null (aPR: 1.43, 95% CI: 0.83, 2.44; Additional file 3). Among unemployed mothers, there was no difference in the prevalence of EBF among mothers in program locations with employment-related breastfeeding laws and mothers in program locations without employment-related breastfeeding laws (aPR: 1.06, 95% CI: 0.64, 1.76; Additional file 3). Among mothers living in program locations with no employment-related breastfeeding laws (Nevada, Pennsylvania, Wisconsin), EBF was 26% less prevalent for employed mothers compared to unemployed mothers (aPR: 0.74, 95% CI: 0.67, 0.83; Additional file 3). There was no difference in the prevalence of EBF among employed mothers in program locations with employment-related breastfeeding laws and unemployed mothers in program locations without employment-related breastfeeding laws (aPR: 1.06, 95% CI: 0.63, 1.78; Additional file 3). There were indications of effect modification by employment status on the relationship between employment-related breastfeeding laws and EBF on both the additive and multiplicative scales, but evidence on the multiplicative scale was stronger (aPR: 1.34, 95% CI: 1.01,1.78; Additional file 3). The estimated effect on the additive scale of employment-related laws among employed mothers was larger than the estimated effect of employment-related laws among unemployed mothers (RERI: 0.25, 95% CI: -0.02,0.53; Additional file 3).

We also observed that mothers who received a WIC breastfeeding consultation had 33% higher prevalence of EBF compared to mothers who did not receive a WIC breastfeeding consultation (aPR: 1.33, 95% CI: 1.05,1.70; Model III; Additional file 4). The association between WIC breastfeeding consultation and EBF was not modified by employment on either the additive or multiplicative scales (Additional file 5).

Our sensitivity analyses on specific individual breastfeeding laws found no strong evidence of association between individual laws and EBF. Specifically, there was no association between mothers in program locations with the law encouraging or requiring employers to provide break time and private space for breastfeeding employees and EBF (aPR: 1.18, 95% CI: 0.72,1.93). There was also no association between mothers in program locations with laws prohibiting employers from discriminating against breastfeeding employees and EBF (aPR: 0.78, 95% CI: 0.61,1.00). Other sensitivity analyses focusing on infant age specific associations showed there was also no relationship between the number of breastfeeding laws and EBF among infants age ranges 0–1 month (aPR: 0.78, 95% CI: 0.61,1.00), or 2–3 months (aPR: 0.98, 95% CI: 0.71,1.36), and 4–5 months (aPR: 1.05, 95% CI: 0.73,1.51). Our sensitivity analysis on the number of law-years found no association between a 10-unit increase in law-years in a program location and EBF (aPR: 1.07, 95% CI: 0.91,1.25). We also conducted sensitivity analyses with mothers’ intention to exclusively breastfeed prior to delivery as a potential confounder and we got similar results.

## Discussion

The objective of this study was to assess the role of breastfeeding laws and programs on EBF among WIC mothers. Our analysis found that the number of laws supporting breastfeeding in a program location was not associated with a WIC mother’s EBF practice. We found that WIC mothers who were employed were less likely to practice EBF compared to WIC mothers without employment in program locations without employment-related breastfeeding laws. However, EBF was more prevalent among employed WIC mothers in workplaces with laws supporting breastfeeding. EBF practice was also more prevalent among mothers who received a WIC breastfeeding consultation compared to mothers who did not receive such a consultation.

Our finding of a higher EBF prevalence among employed mothers working in program locations with employment-related breastfeeding laws may lend support to the important role laws supporting breastfeeding at the workplace can have at promoting breastfeeding practice. The finding of no strong association between the number of laws supporting breastfeeding and EBF practice among all mothers could be due to several reasons. Most states’ laws supporting breastfeeding lacked enforcement provisions and few of these laws have penalties for violations [11]. Enforcing laws supporting breastfeeding without penalties for violations or incentives to encourage compliance may render laws supporting breastfeeding less effective [11]. Another possible reason for our observed finding is that mothers may be unaware of laws supporting breastfeeding and therefore could not take advantage of such laws to breastfeed [28]. Instances have been reported of unawareness of the existence of laws permitting mothers to breastfeed in any public or private location, and where mothers were told to stop breastfeeding or leave the vicinity of premises [29]. Mothers in such situations may stop breastfeeding because they feel embarrassed and afraid of being stigmatized by the people around them [30, 31]. Another plausible explanation for our finding is that some of these laws only encourage, but do not require employers to provide specific breastfeeding protections [11]. The type, size, or both of the workplace may also make it difficult for employers to comply with laws that encourage or require employers to provide break time and private space for breastfeeding employees [10]. It has also been reported that some employers do not adhere to states’ laws supporting breastfeeding at the workplace because they are unaware of the existence of such laws [10]. Our finding could also be due to the lack of variation in distribution of the number of breastfeeding laws and program locations in our study. Our observed finding may also be due some working mothers complementing partial breastfeeding with infant formula.

The finding on EBF, which was more prevalent among mothers who received a WIC breastfeeding consultation compared to mothers who did not receive a WIC breastfeeding consultation, was not surprising. Mothers who received breastfeeding support from either WIC staff [32], lactation consultants [32, 33] or peer counselors [32–34] have reported increased breastfeeding rates. Our finding is a reflection that WIC breastfeeding consultation is beneficial to WIC mothers; therefore, its practice may be encouraged. In theory, all mothers should have received WIC consultation related to breastfeeding. It’s unclear why many mothers did not report receiving breastfeeding information.

Our study also showed that among WIC mothers in program locations without employment-related breastfeeding laws, those who were employed were less likely to practice EBF compared to those without employment. Being employed has been reported as a barrier to EBF in the US, but among the general population [6, 35]. The lower prevalence of EBF among employed compared to unemployed mothers might be because breastfeeding is time intensive and requires more time than mixed or formula only feeding [7]. It could also be due to the separation of infants from their mothers among employed mothers during working hours compared to unemployed mothers [36]. In addition, workplace policies, such as short or unpaid maternity leaves could negatively impact EBF practice among employed mothers [37, 38]. One other possible reason for our observed finding could be that some breastfeeding mothers may not be supported to breastfeed at the workplace [39].

Contrary to previous studies that were not restricted to WIC mothers [40, 41], our study found that primiparous mothers were more likely to practice EBF compared to multiparous mothers. Our finding may be because of our cohort of WIC mothers may be different in some ways than the general population. WIC mothers are more likely to be unemployed and receive a WIC breastfeeding consultation compared to the general population. Our findings on the lower prevalence of EBF among mothers who smoked and had a Cesarean section align with published literature [42, 43]. The higher prevalence of EBF among mothers with a previous history of breastfeeding compared to mothers without such history has also been reported [44]. Consistent with many other studies [45–47], EBF in our study was also more prevalent among Caucasian/White mothers compared to Hispanic/Latina mothers. Our finding on EBF, which was more prevalent among mothers who received breastfeeding information from a breastfeeding support group compared to mothers who did not receive such support, has also been reported [48].

This study had strengths and limitations that should be acknowledged. Our study is unique as it is the first study to examine the role of breastfeeding laws and programs among WIC participants in the US. Our findings may not be generalizable to the general population as it was restricted to WIC participants in seven program locations. Nevertheless, our findings fill an important gap in the literature as it provides insight on the role of breastfeeding laws and programs among WIC mothers in the US. A major limitation of our study is that the design does not allow for our findings to infer causality. Another limitation is that we did not have individual-level data, particularly among employed mothers on whether their employers implemented laws supporting breastfeeding at their workplace. Additionally, some of our predictor variables were self-reported, but we have no reason to expect recall bias to be different between mothers who practiced EBF and those who did not. Another limitation was that we could not control for some potential confounders such as the maternity leave status of employed and medical conditions (e.g., a mother taking certain medications, human immunodeficiency virus (HIV), active tuberculosis, brucellosis, or varicella), which are associated with breastfeeding [49, 50], but these variables were not collected. In addition, the sample size for many of the program locations in our study were small and therefore our estimates may have been subject to random error.

## Conclusions

We found that mothers who received a WIC breastfeeding consultation were more likely to practice EBF compared to mothers who did not receive such a consultation. While we also found that women who were employed were less likely to practice EBF, our study lends support to laws supporting breastfeeding at the workplace as these laws can promote EBF among employed mothers. We recommend for further research using large prospective studies to assess specific breastfeeding laws on EBF in all states.

## Supplementary Information

**Additional file 1.** Specific breastfeeding laws and the years of enactment in seven program locations.

**Additional file 2.** List of potential confounders adjusted for in each model.

**Additional file 3.** Effect modification of the association between employment-related breastfeeding laws and EBF by employment status (model II*).

**Additional file 4.** The relationship between WIC breastfeeding consultation and EBF (Model III*).

**Additional file 5.** Effect modification of the association between WIC breastfeeding consultation and EBF by employment status (Model IV*).

## Data Availability

Data is available on reasonable request.

## Abbreviations

aPR: Adjusted prevalence ratio
EBF: Exclusive breastfeeding
ITO: Indian Tribal Organization
NCSL: National Conference of State Legislatures
RERI: Relative excess risk due to interaction
SD: Standard deviation
US: United States
WIC: Women, infants, and children
