# Supplementary material for: The role of state breastfeeding laws and programs on exclusive breastfeeding practice among mothers in the special supplemental nutrition program for Women, Infants, and Children (WIC)
Source: Int Breastfeed J. 2022 Jun 25;17:46. doi: 10.1186/s13006-022-00490-9 (PMC9233787; doi:10.1186/s13006-022-00490-9)
Supplement: Supplementary file 1 — Additional file 1. Specific breastfeeding laws and the years of enactment in seven program locations. [file 13006_2022_490_MOESM1_ESM.docx]

Additional file 1. Specific breastfeeding laws and the years of enactment in seven program locations

| State | Employers encouraged or required to provide break time and private space | Employers prohibited from discriminating against breastfeeding employees | Breastfeeding permitted in any public or private location | Breastfeeding exempt from public indecency laws | Breastfeeding mothers exempt from jury duty | Any employment-related laws ** (%) | Total number of laws | Number of law-years*** |
| --- | --- | --- | --- | --- | --- | --- | --- | --- |
| CN | 2006 | Nil* | 2004 | 2004 | 2004 | Yes | 4 | 62 |
| GA | 1999 | Nil | 1999 | Nil | Nil | Yes | 2 | 42 |
| MA | Nil | 2018 | 2008 | 2008 | Nil | Yes | 3 | 26 |
| NV | Nil | Nil | 1995 | 1995 | Nil | No | 2 | 50 |
| PA | Nil | Nil | 2007 | 2007 | 2015 | No | 3 | 31 |
| WI | Nil | Nil | 2009 | 1995 | Nil | No | 2 | 36 |
| WY | 2003 | Nil | 2007 | 2007 | Nil | Yes | 3 | 43 |

CN=Chickasaw Nation; GA= Georgia; MA= Massachusetts; NV=Nevada; PA= Pennsylvania; WI=Wisconsin; WY= Wyoming

*Discussion with program staff from CN indicates that employers are encouraged not to discriminate.

**Employment-related laws are laws that require or encourage employers to provide break time and private space

for breastfeeding or laws that prohibit employers from discriminating against breastfeeding employees.

***Number of law-years is the number of total years of laws enacted in each state.
