# Supplementary material for: The role of state breastfeeding laws and programs on exclusive breastfeeding practice among mothers in the special supplemental nutrition program for Women, Infants, and Children (WIC)
Source: Int Breastfeed J. 2022 Jun 25;17:46. doi: 10.1186/s13006-022-00490-9 (PMC9233787; doi:10.1186/s13006-022-00490-9)
Supplement: Supplementary file 2 — Additional file 2. List of potential confounders adjusted for in each model. [file 13006_2022_490_MOESM2_ESM.docx]

Additional file 2. List of potential confounders adjusted for in each model

| Model I | Model II | Model III | Model IV |
| --- | --- | --- | --- |
| WIC breastfeeding consultation | Number of breastfeeding laws | Number of breastfeeding laws | Number of breastfeeding laws |
| Has an adult support for baby at home | WIC breastfeeding consultation | Has an adult support for baby at home | Has an adult support for baby at home |
| Current smoker | Has an adult support for baby at home | Current smoker | Current smoker |
| Parity | Current smoker | Parity | Parity |
| Infant was delivered by Cesarean section | Parity | Infant was delivered by Cesarean section | Infant was delivered by Cesarean section |
| Infant was born premature | Infant was delivered by Cesarean section | Infant was born premature | Infant was born premature |
| Received breastfeeding promotional messages | Infant was born premature | Received breastfeeding promotional messages | Received breastfeeding promotional messages |
| Previous history of breastfeeding | Received breastfeeding promotional messages | Previous history of breastfeeding | Previous history of breastfeeding |
| Received breastfeeding information from any breastfeeding support group | Previous history of breastfeeding | Received breastfeeding information from any breastfeeding support group | Received breastfeeding information from any breastfeeding support group |
| Mother's age (years) | Received breastfeeding information from any breastfeeding support group | Mother's age (years) | Mother's age (years) |
| Infant’s age | Mother's age (years) | Infant’s age | Infant’s age |
| Education | Infant’s age | Education | Education |
| Marital status | Education | Marital status | Marital status |
| Race/Ethnicity | Marital status | Race/Ethnicity | Race/Ethnicity |
|  | Race/Ethnicity | Employed |  |
