# Supplementary material for: The role of state breastfeeding laws and programs on exclusive breastfeeding practice among mothers in the special supplemental nutrition program for Women, Infants, and Children (WIC)
Source: Int Breastfeed J. 2022 Jun 25;17:46. doi: 10.1186/s13006-022-00490-9 (PMC9233787; doi:10.1186/s13006-022-00490-9)
Supplement: Supplementary file 3 — Additional file 3. Effect modification of the association between employment-related breastfeeding laws and EBF by employment status (model II*). [file 13006_2022_490_MOESM3_ESM.docx]

Additional file 3. Effect modification of the association between employment-related breastfeeding laws and EBF by employment status (model II*).

|  | No employment-related breastfeeding law | |  | Has employment-related breastfeeding laws | |  |
| --- | --- | --- | --- | --- | --- | --- |
|  | N with/without outcome | PR (95% CI) |  | N with/without outcome | PR (95% CI) | PR (95% CI) comparing EBF in program locations with employment-related breastfeeding laws vs. not within strata of employment status |
| No employment | 202/338 | 1.00 (Reference) |  | 89/143 | 1.06 (0.64,1.76) *p=0.810* | 1.06 (0.64,1.76) *p=0.810* |
| Employed | 58/165 | 0.74 (0.67,0.83) *p<0.001* |  | 43/76 | 1.06 (0.63,1.78) *p=0.826* | 1.43 (0.83, 2.44) *p=0.196* |

Measure of effect modification on additive scale: Relative excess risk due to interaction [RERI] (95% CI) = 0.25 (-0.02,0.53); p=0.073

Measure of effect modification on multiplicative scale: ratio of PRs (95% CI) = 1.34 (1.01,1.78); p=0.042

*Model II: Prevalence ratios (PRs) are adjusted for number of breastfeeding laws, WIC breastfeeding consultation and individual-and program level factors as in Model I.
