# Supplementary material for: The role of state breastfeeding laws and programs on exclusive breastfeeding practice among mothers in the special supplemental nutrition program for Women, Infants, and Children (WIC)
Source: Int Breastfeed J. 2022 Jun 25;17:46. doi: 10.1186/s13006-022-00490-9 (PMC9233787; doi:10.1186/s13006-022-00490-9)
Supplement: Supplementary file 4 — Additional file 4. The relationship between WIC breastfeeding consultation and EBF (Model III*). [file 13006_2022_490_MOESM4_ESM.docx]

Additional file 4. The relationship between WIC breastfeeding consultation and EBF (Model III*).

| Variable | **Adjusted PR (95% CI) |
| --- | --- |
| **Main exposure of interest** |  |
| No | Reference |
| WIC breastfeeding consultation | 1.33 (1.05,1.70) |
| **Potential confounders** |  |
| No | Reference |
| Employed | 0.83 (0.71,0.98) |
| No | Reference |
| Has an adult support for baby at home | 1.02 (0.87,1.19) |
| No | Reference |
| Current smoker | 0.62 (0.45,0.86) |
| Parity |  |
| multiparity | Reference |
| primiparity | 2.13 (1.42,3.19) |
| Vaginal delivery | Reference |
| Cesarean section | 0.80 (0.74,0.87) |
| Term baby | Reference |
| Premature baby | 0.83 (0.61,1.11) |
| No | Reference |
| Received breastfeeding promotional messages | 1.05 (0.81,1.37) |
| No | Reference |
| Previous history of breastfeeding | 2.68 (1.85,3.88) |
| No | Reference |
| Received breastfeeding information from a breastfeeding support group | 1.30 (1.11,1.52) |
| Mother's age (years) | 1.00 (0.99,1.01) |
| Infant’s age |  |
| < 8 weeks | Reference |
| 8-16 weeks | 1.11 (0.96,1.29) |
| > 16 weeks | 1.08 (0.93,1.25) |
| Education |  |
| < High school graduate | Reference |
| ≥ High school graduate | 1.02 (0.72,1.43) |
| Marital status |  |
| Married/living with partner | Reference |
| Single | 0.95 (0.83,1.08) |
| Race/Ethnicity |  |
| Caucasian/White | Reference |
| Asian/Pacific Islander | 0.68 (0.37,1.25) |
| Black/African American | 0.81 (0.59,1.13) |
| American Indian/Alaska Native | 1.03 (0.79,1.34) |
| Hispanic/Latina | 0.64 (0.45,0.92) |
| Multiracial | 0.82 (0.52,1.29) |
| Other | 2.07 (0.85,5.05) |
| Number of breastfeeding laws | 0.89 (0.72,1.09) |

*Model III adjusted for number of breastfeeding laws and various individual and program level factors

**Adjusted PR is adjusted prevalence ratio
