# Supplementary material for: The role of state breastfeeding laws and programs on exclusive breastfeeding practice among mothers in the special supplemental nutrition program for Women, Infants, and Children (WIC)
Source: Int Breastfeed J. 2022 Jun 25;17:46. doi: 10.1186/s13006-022-00490-9 (PMC9233787; doi:10.1186/s13006-022-00490-9)
Supplement: Supplementary file 5 — Additional file 5. Effect modification of the association between WIC breastfeeding consultation and EBF by employment status (Model IV*). [file 13006_2022_490_MOESM5_ESM.docx]

Additional file 5. Effect modification of the association between WIC breastfeeding consultation and EBF by employment status (Model IV*)

|  | No WIC breastfeeding consultation | |  | WIC breastfeeding consultation | |  |
| --- | --- | --- | --- | --- | --- | --- |
|  | N with/without outcome | PR (95% CI) |  | N with/without outcome | PR (95% CI) | PR (95% CI) comparing EBF in WIC breastfeeding consultation vs. not within strata of employment status |
| No employment | 31/73 | 1.00 (Reference) |  | 257/399 | 1.30 (1.09,1.56) *p=0.003* | 1.30 (1.09,1.56) *p=0.003* |
| Employed | 9/33 | 0.77 (0.49,1.22) *p=0.262* |  | 92/207 | 1.10 (0.90,1.34) *p=0.364* | 1.43 (0.88,2.31) *p=0.149* |

Measure of effect modification on additive scale: Relative excess risk due to interaction [RERI] (95% CI) = 0.02 (-0.22,0.27); p=0.890

Measure of effect modification on multiplicative scale: ratio of PRs (95% CI) =1.09 (0.76,1.58) p=0.634

*Model IV: Prevalence Ratios (PRs) are adjusted for individual and program level factors including the number of breastfeeding laws as described in the text above
